# Genotyping of *TRIM5 *locus in northern pig-tailed macaques (*Macaca leonina*), a primate species susceptible to Human Immunodeficiency Virus type 1 infection

**DOI:** 10.1186/1742-4690-6-58

**Published:** 2009-06-09

**Authors:** Yi-Qun Kuang, Xia Tang, Feng-Liang Liu, Xue-Long Jiang, Ya-Ping Zhang, Guangxia Gao, Yong-Tang Zheng

**Affiliations:** 1 Key Laboratory of Animal Models and Human Disease Mechanisms, Kunming Institute of Zoology, Chinese Academy of Sciences, Kunming, Yunnan 650223, PR China; 2 State Key Laboratory of Genetic Resources and Evolution, Kunming Institute of Zoology, Chinese Academy of Sciences, Kunming, Yunnan 650223, PR China; 3 Institute of Biophysics, Chinese Academy of Sciences, Beijing 100101, PR China; 4 Graduate School of Chinese Academy of Sciences, Beijing 100039, PR China

## Abstract

**Background:**

The pig-tailed macaques are the only Old World monkeys known to be susceptible to human immunodeficiency virus type 1 (HIV-1) infection. We have previously reported that the *TRIM5-Cyclophilin A *(*TRIMCyp*) fusion in pig-tailed macaques (*Macaca nemestrina*) is dysfunctional in restricting HIV-1, which may explain why pig-tailed macaques are susceptible to HIV-1 infection. Similar results have also been reported by other groups. However, according to the current primate taxonomy, the previously reported *M. nemestrina *are further classified into three species, which all belong to the *Macaca spp*. This calls for the need to look into the previous studies in more details.

**Results:**

The local species Northern pig-tailed macaque (*M. leonina*) was analyzed for the correlation of *TRIM5 *structure and HIV-1 infection. Eleven *M. leonina *animals were analyzed, and all of them were found to possess *TRIM5-CypA *fusion at the *TRIM5 *locus. The transcripts encoding the dysfunctional *TRIM5-CypA *should result from the G-to-T mutation in the 3'-splicing site of intron 6. Polymorphism in the putative TRIMCyp recognition domain was observed. The peripheral blood mononuclear cells (PBMCs) of *M. leonina *were susceptible to HIV-1 infection. Consistent with the previous results, expression of the *M. leonina *TRIMCyp in HeLa-T4 cells rendered the cells resistant to HIV-2_ROD _but not to SIVmac239 infection.

**Conclusion:**

The susceptibility of *M. leonina *to HIV-1 infection is due to the dysfunctional *TRIM5-CypA *fusion in the *TRIM5 *locus. This finding should broaden our perspective in developing better HIV/AIDS non-human primate animal models.

## Background

Human immunodeficiency virus type 1 (HIV-1) originated from cross-species transmission from chimpanzees to humans and is the major causative agent of human acquired immunodeficiency syndrome (AIDS) pandemic [1-3]. Although HIV-1 infects human CD4^+ ^cells, it does not infect most non-human primates (NHP). Studies using Vesicular Stomatitis virus G-glycoprotein (VSV-G) pseudotyped viruses, which bypass the receptor restriction, revealed that species-specific host factors restrict HIV-1 infection [4]. For example, the host restriction factor tripartite motif protein 5α (TRIM5α) potently blocks HIV-1 replication in rhesus macaque (*M. mulatta*) through species-specific post-entry restriction in Old World monkeys [5].

TRIM5α is a member of the TRIM family, which contains the RING, B-Box2 and coiled-coil domains, and a C-terminal B30.2/SPRY domain. TRIM5α interacts with the capsid (CA) portion of HIV-1 Gag protein through its B30.2/SPRY domain, which determines the specificity and potency of TRIM5α restriction to retroviruses [5,6]. Host protein cyclophilin A (CypA) interacts with the CA through incorporation into HIV-1 particles, and modulates HIV-1 replication in host cells [7-9]. It has been documented that in Old World monkey cells, CypA is required for TRIM5α-mediated resistance to HIV-1 [10]. The New World primate owl monkey (*Aotus*) expresses a TRIM5-CypA (TRIMCyp) fusion protein, in which the B30.2/SPRY domain of TRIM5α is replaced by CypA resulting from retrotransposition of the *CypA *pseudogene cDNA into the seventh intron at the *TRIM5 *locus. The owl monkey TRIM5-CypA (omTRIMCyp) restricts several retroviruses including HIV-1, simian immunodeficiency virus (SIV) and feline immunodeficiency virus (FIV) [11,12]. Recently, we and others reported that in pig-tailed macaques the B30.2/SPRY domain is replaced by retrotransposed *CypA *in the 3'-UTR of *TRIM5 *in a fashion different from that in the owl monkey, resulting in the failure of restriction to HIV-1 replication in pig-tailed macaques [13-17].

According to the current widely-accepted primate taxonomy based on more morphological studies and phylogeographic analyses, the previously reported *Macaca nemestrina *group is divided into three species: Sunda pig-tailed macaque (*M. nemestrina*), Northern pig-tailed macaque (*M. leonina*), and Mentawai macaque (*M. pagensis*) [18-21]. The *M. nemestrina *distributes in Malay Peninsula from about 7°30'N, Sumatra, Bangka and Borneo. The *M. leonina *ranges from about 8°N in Peninsular Thailand, through Burma and Indochina into Bangladesh, India extending north as far as to the Brahmaputra, and the southernmost Yunnan, China. The *M. pagensis *locates in the Mentawai islands [18]. The previously studied pig-tailed macaques may contain individuals of different species. Here, we analyzed the susceptibility of the local species *M. leonina *in Yunnan to HIV-1 infection and the *TRIM5 *locus. The fusion pattern of TRIMCyp and the polymorphism of the TRIMCyp recognition domain in *M. leonina *were characterized.

## Results

### Characterization of the TRIMCyp fusion gene in *M. leonina*

**Figure 1:**
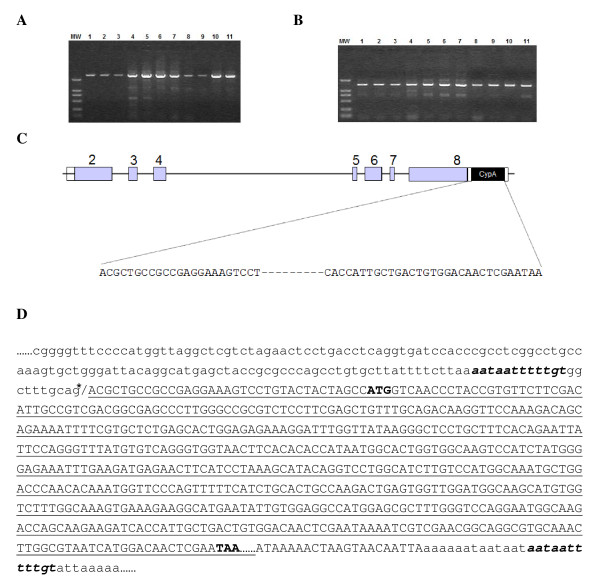
**Formation of TRIMCyp fusion gene in *M. leonina***. (A and B) The genomic sequences spanning from the 5' end of exon 8 to the 3' end of exon 8 (A) or to the 3'-UTR of *CypA *cDNA (B) were PCR amplified and subject to electrophoresis analysis. MW: DNA molecular weight marker DL-2000; Lane 1–11: *M. leonina *samples 524, 528, 551, KMZ-1, KMZ-2, KMZ-4, KMZ-5, 87015, 93201, 97203, and 99201. (C) Schematic structure of the *TRIM5Cyp *fusion. The exons are represented as boxes with the coding region being shaded, and the sequence of the inserted *CypA *cDNA is denoted below. (D) The *CypA *pseudogene cDNA retrotransposed into *TRIM5 *locus. The asterisk (*) indicates the splicing acceptor, *CypA *pseudogene cDNA sequence is underlined, target site duplication (TSD) is in bold italic, and the start or stop codon of inserted CypA cDNA is in bold-type.

To investigate the correlation between the TRIM5α sequence and the susceptibility to infection by HIV-1 in *M. leonina*, the genomic sequence of the *TRIM5 *locus of 11 animals from several different populations was analyzed (Table 1). A pair of specific PCR primers was designed based on the human *TRIM5 *genomic sequence, with the forward primer in the *TRIM5 *exon 8 and the reverse primer in the adjacent genomic region after *TRIM5 *3'-UTR (Table 2). A fragment of about 2, 800 bp was amplified (Fig. 1A), indicating that the *TRIM5 *locus is longer than normal and thus the *TRIM5-CypA *pattern might exist. To confirm this notion, another pair of primers was designed, with the forward one in exon 8 and the reverse one in the *CypA *sequence (Table 2). Indeed, a *CypA *cDNA sequence is inserted in the *TRIM5 *locus in all *M. leonina *(Fig. 1B, C), which disrupts the normal *TRIM5*.

**Table 1:** The information of *M. leonina *samples used in this study.

**Sample Number #**	**Sex**	**Weight**	**Origin of Macaque**	**Sampling Time**	**Population Location**
524	Male	ND	Yunnan, China	1998-4-15	KIZ, CAS
528	Female	ND	Yunnan, China	1988-4-5	KIZ, CAS
551	Female	ND	Yunnan, China	2000-11	KIZ, CAS
87015	Male	11 kg	Yunnan, China	2008-4-11	KIZ, CAS
93201	Male	12 kg	Yunnan, China	2008-4-11	KIZ, CAS
97203	Male	9.5 kg	Yunnan, China	2008-4-11	KIZ, CAS
99201	Male	10 kg	Yunnan, China	2008-4-11	KIZ, CAS
KMZ-1	Male	14.5 kg	Yunnan, China	2008-3-28	KMZ
KMZ-2	Male	ND	Yunnan, China	2008-3-28	KMZ
KMZ-4	Female	6.4 kg	Yunnan, China	2008-3-28	KMZ
KMZ-5	Male	ND	Yunnan, China	2008-3-28	KMZ

ND: not determined

**Table 2:** Primers used for genomic and RT-PCR amplification.

**No**.	**Primer Name **	**Sequence (5' – 3')**
1	T5in6F1	TGGAATTCATGTGGTGTCAGGGTG
2	T5in7F1	CAGCTACCCTGTGGCTTATCAT
3	T5in7R1	GACTTGAGAGAAAGCTGGGAGGA
4	T5ex8F1	CTGGCTCCAAACAACATTTC
5	T5ex8F2	TGACTCTGTGCTCACCAAGCT
6	T5ex8R1	ATATATAGAAGGCAGAATTGAAG
7	T5ex8R2	TCAAGAGCTTGGTGAGC
8	T5ex8R3	AGCCCAGGACGCCAGTACAATA
9	CypAR	TTATTCGAGTTGTCCAC
10	TRIMCypF	ATGGCTTCTGGAATCCTGGTTAATGTAAAG
11	TRIMCypR	CTATTCGAGTTGTCCACAGTCAGCAAT

To further characterize the *CypA *insertion in the *TRIM5 *locus, we performed several other PCR reactions with different pairs of primers (Table 2). The PCR products were recovered and sequenced. The sequencing results revealed that the *CypA *pseudogene cDNA insertion in the *TRIM5 *locus resulted from a LINE (long interspersed nuclear element)-1-mediated retrotransposition (Fig. 1D), which is very common in mammals [22,23].

### Expression of TRIMCyp fusion gene in *M. leonina*

To test whether the *TRIMCyp *fusion gene in *M. leonina *is transcribed to produce mature transcripts, the RNAs from 8 *M. leonina *samples were reverse transcribed and PCR amplified using specific primers TRIMCypF and TRIMCypR (Table 2). Multiple mature *TRIMCyp *transcripts with different lengths were detected in eight *M. leonina *samples, but not in the Chinese rhesus macaque samples (data not shown). Sequencing analysis of the PCR products revealed that both exon 7 and exon 8 were spliced out in all major isoforms leaving exon 6 fused to the *CypA *cDNA in frame (data not shown), as previously reported [13-16].

**Figure 2:**
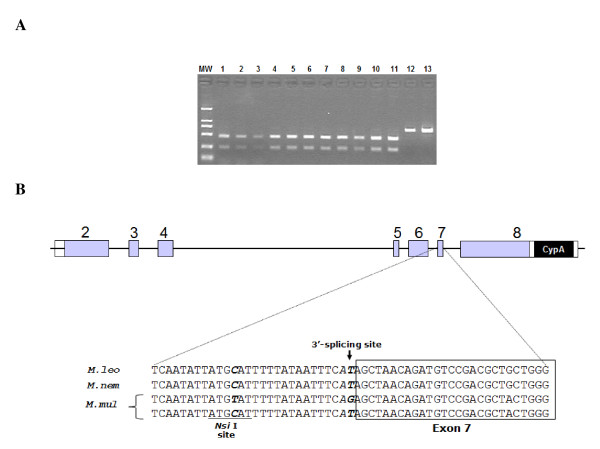
**Analysis of the 3'-splicing site in intron 6 at the *TRIM5 *locus**. (A) The sequences encompassing the 3'-splicing site in intron 6 were PCR amplified from the genomic DNA of the following samples. The PCR products were digested with restriction endonuclease *Nsi *I, followed by electrophoresis in a 1.4% agarose gel. MW: molecular weight DNA marker DL-2000; Lane 1–11: *M. leonina *samples as described in the legend to figure 1B; Lane12: *M. mulatta *95005 PBMCs; Lane 13: a *M. mulatta *immortalized B cell line. (B) Schematic representation of the position and sequences of the 3'-splicing acceptor site in intron 6. The boxes represent the exons of the *TRIM5 *genome, and the lines represent the introns. The 3'-splicing site is indicated by the arrow, and the *Nsi *I recognition sequence is underlined.

To understand why exons 7 and 8 were not included in the mature transcripts, the sequences of introns 6 and 7 were analyzed for aberrant splicing sites. The *Nsi *I restriction site upstream the 3' splicing site of intron 6 has been reported to be closely linked to the mutation within the site [17], which allowed a convenient screening of the G-to-T substitution in the splicing site (Fig. 2B). Analysis of the PCR product flanking intron 6 by the *Nsi *I restriction digestion revealed that all *M. leonina *were homozygous for the *Nsi *I site (Fig. 2A). The G-to-T substitution in the 3' splicing site of intron 6 was confirmed by sequencing analysis of the PCR products in all the *M. leonina *samples (Fig. 2B). The G-to-T substitution in the 3' splicing site of intron 6 should prevent the inclusion of exon 7 during splicing. Sequencing analysis revealed that the 3' splicing site of intron 7 was normal (data not shown). The exclusion of exon 8 in the mature transcript is likely the result of alternative splicing, as previously observed [13].

### Polymorphism analysis in the TRIMCyp recognition domain

**Figure 3:**
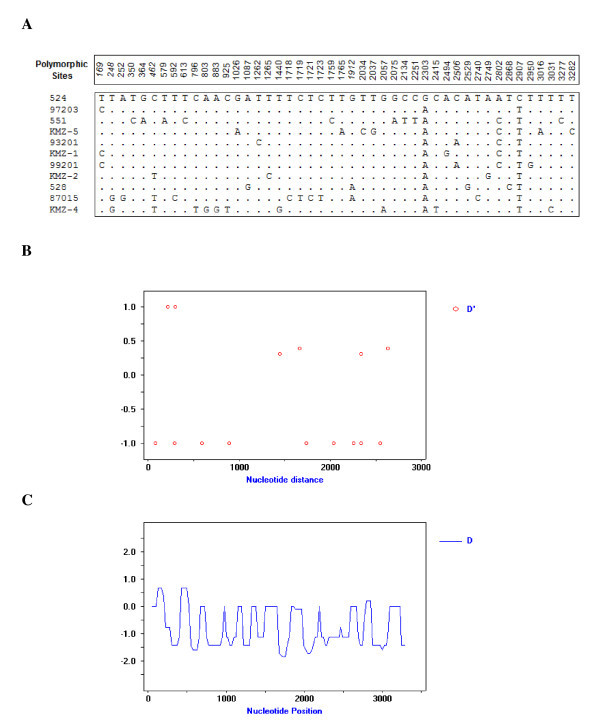
**Genetic polymorphism analysis of the TRIMCyp recognition domain**. (A) Polymorphic sites of the nucleotide sequence of the TRIMCyp recognition domain. The boxed numbers indicate the polymorphic sites, with 6 Parsimony informative positions in italic. The boxed sequences showed the Haplotype of the *M. leonina *samples. Dots present the identical nucleotides. (B) The linkage disequilibrium analysis of all sequenced sites. The histogram X axis plots the D' value, the Y axis plots the DNA sequence nucleotide positions. (C) Tajima's or Fu and Li's D test of the total number of mutations for neutrality test through DnaSP program.

A fragment of 3348 bp from intron 6 to the 3' genomic adjacent region was analyzed for polymorphisms via DnaSP 4.5 program [24,25]. In the 11 Northern pig-tailed macaques, a total of 46 polymorphic nucleotide sites were identified, including 40 Singleton variable sites and 6 Parsimony informative sites (Fig. 3A). Among these sites, 16 sites (site 579, 592, 613, 796, 803, 883, 925, 1026, 1087, 2134, 2251, 2303, 2415, 2494, 2506 and 2529) are in the coding region (39%), and the others (site 169, 248, 252, 350, 364, 462, 1262, 1265, 1440, 1718, 1719, 1721, 1723, 1759, 1765, 1912, 2034, 2037, 2057, 2075, 2740, 2749, 2802, 2868, 2907, 2950, 3016, 3031, 3277 and 3282) are in the noncoding region. There are 15 nonsynonymous variation sites in the coding region, except for the synonymous site 2303. In KMZ-4, we identified an insertion-mutation, which results in frame shift relative to the coding region (data no shown). Next, we sought to determine whether the observed polymorphisms are consonant with the real status. Linkage disequilibrium (LD) describes a situation in which some combinations of alleles or genetic markers occur more or less frequently in a population than would be expected from a random formation of haplotypes from alleles based on their frequencies. The occurrence of LD permits the construction of Haplotype. The LD analysis (Fisher's exact test and Chi-square test) showed that no recombinant event occurred, and the degree of LD is strong (P < 0.001) (Fig. 3B). Additionally, the Neutrality theory was used to detect the intra-specific polymorphism level, and two approaches (Tajama's D test and Fu and Li's D test) based on different algorithm models were employed. The Neutrality test of Tajama's D test demonstrated no statistical significance in polymorphism along the sequences (P < 0.005), while Fu and Li's D test were significant (P < 0.005) (Fig. 3C).

**Figure 4:**
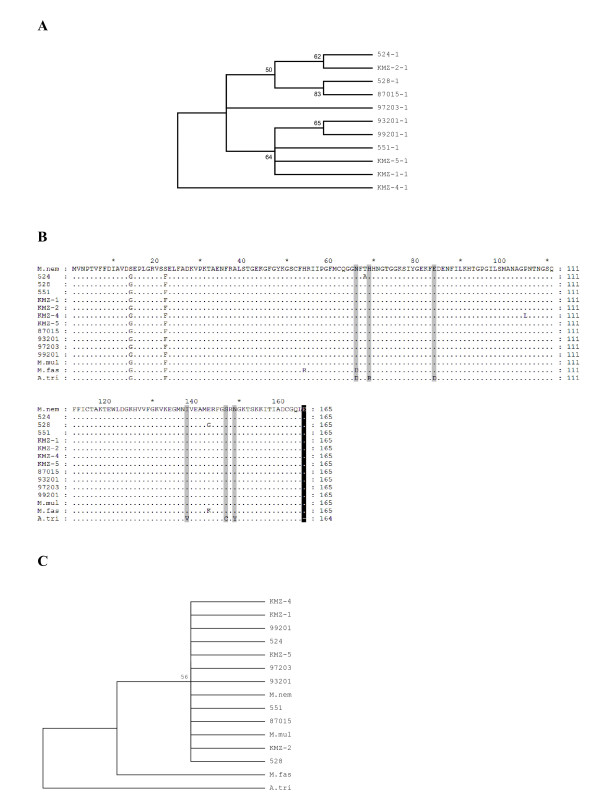
**Phylogenetic analysis of primate TRIMCyp recognition domain**. (A) Neighbor-joining nucleotide tree of the 11 Northern pig-tailed macaques based on TRIMCyp. Bootstrap values are based on 1,000 replicates. (B) Alignment of putative CypA amino acid sequences of primate TRIMCyp. The gray bar indicates mutation sites in Old World primates, and the bold bar indicates deletion mutations, as compared to *A. trivirgatus*. (C) Phylogenetic analysis of the primate TRIMCyp recognition domain (based on CypA amino acid sequence) with the *A. trivirgatus *as outgroup. Bootstrap values are based on 1,000 replicates.

The coding sequences of *TRIMCyp *exon 7, exon 8 and *CypA *from *M. leonina *were assembled to deduce the putative amino acid sequences. The phylogenetic tree based on the putative amino acid sequences demonstrated that the 11 *M. leonina *are divided into several major subgroups (Fig. 4A), which may explain the high polymorphic in this region. In the *Aotus *and *Macaca *TRIMCyp proteins, the CypA part is the recognition domain mediating the binding of the fusion protein to the incoming viral capsids. The putative amino acid sequences of the CypA domain from various species were aligned. No major differences were found in sequences of CypA inserted in TRIMCyp from diverse *M leonina*. The results clearly show that the sequences are homologous among the Old World macaque *M. leonina*, *M. nemestrina *and *M. mullata *species, while the *M. fasciculari *and the New World monkey *A. trivirgatus *were much less homologous (Fig. 4B). In addition, some amino acids critical for the restriction of HIV-1 in *A. trivirgatus*, such as N66 and H69, were observed in *M. leonina*. The phylogenetic tree among these primates was constructed based on the inserted CypA amino acid sequences. The result revealed that the *Macaca spp *species members *M. leonina*, *M. nemestrina *and *M. mulatta *were relatively close in homology, the *M. fasciculari *and the New World monkey *A. trivirgatus *were in two obviously more distant groups (Fig. 4C).

### Susceptibility of *M. leonina *PBMCs to HIV-1 infection

**Figure 5:**
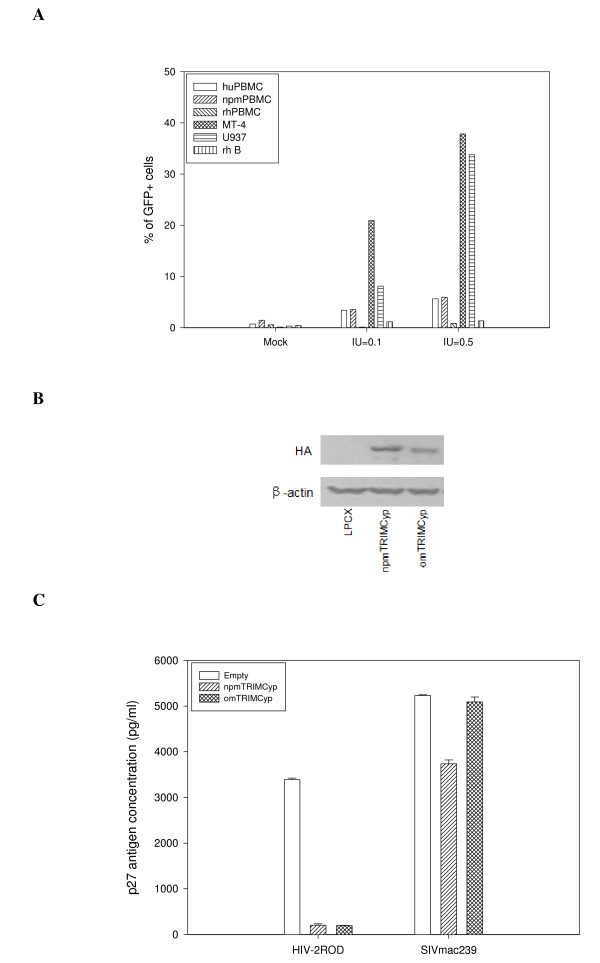
**Restriction activities of *M. leonina *TRIMCyp on lentiviruses**. (A) The indicated cells were infected with HIV-GFP-VSVG at the indicated infection unit (IU). IU = 0.1 or 0.5 denotes 10-fold or 2-fold dilution of HIV-GFP-VSVG viral stocks, respectively. Percentage of infected cells was counted 48 hours later by FACS analysis of GFP positive cells. The result is representative of three independent experiments. npmPBMC: northern pig-tailed macaque PBMC; huPBMC: human PBMC; rhPBMC: rhesus PBMC; MT-4: human T cell line MT-4; U937: human monocyte line U937; rh B: a Chinese rhesus macaque transformed B immortalized cell line. (B) The HA-tagged TRIMCyp cloned from northern pig-tailed macaque and owl monkey TRIMCyp were stably expressed in HeLa-T4 cells. The expression of the proteins was confirmed by Western blotting. (C) The cells were infected with HIV-2_ROD _or SIVmac239 virus at MOI (multiplicity of infection) = 0.02. Four days post-infection, the capsid p27 antigen levels in the culture supernatants were measured by quantitative ELISA assays. The result is representative of three independent experiments.

To determine the susceptibility of *M. leonina *to infection by HIV-1, the PBMCs were isolated from EDTA K2-treated whole blood. The PBMCs from *M. mulatta*, which are known to be resistant to HIV-1 infection, were used as a negative control, and the PBMCs from human, which are known to be susceptible to HIV-1 infection, were used as a positive control. The susceptible human T cell line MT-4, the monocyte cell line U937, and the resistant Chinese rhesus macaque transformed B lymphocyte cell line were also used as controls. The cells were challenged with VSV-G pseudotyped HIV-1 carrying the GFP reporter (HIV-GFP-VSVG) at the infection unit (IU) of 0.1 and 0.5. The percentage of GFP positive cells counted by FACS analysis was used as an indicator for the susceptibility of the cells to HIV-1 infection. As expected, the *M. mulatta *PBMCs were relatively resistant to HIV-1 infection (Fig. 5A). In comparison, the *M. leonina *PBMCs demonstrated a magnitude of susceptibility to HIV-GFP-VSVG infection comparable to the human PBMCs (Fig. 5A). These results established that the *M. leonina *are susceptible to HIV-1 infection.

To test whether the *M. leonina *TRIMCyp can restrict other retroviruses, we generated HeLa-T4 cell lines expressing C-terminally HA-tagged TRIMCyp of *M. leonina *and *A. trivirgatus*. The expression of the TRIMCyp fusion proteins (npmTRIMCyp for northern pigtailed macaque TRIMCyp and omTRIMCyp for owl monkey TRIMCyp) was confirmed by Western immunoblotting (Fig. 5B). The cells were challenged with HIV-2_ROD _(MOI = 0.02). Replication of the virus in these cells was evaluated by measuring the capsid p27 antigen levels. The results demonstrated that both npmTRIMCyp and omTRIMCyp actively restricted HIV-2_ROD _by about 16-fold (Fig. 5C). The cells were also assayed for their restriction to SIVmac239 replication. The npmTRIMCyp demonstrated very moderate restriction activity, while the omTRIMCyp could not inhibit SIVmac239 replication (Fig. 5C).

## Discussion

Because of their close evolutionary relationship to humans, NHPs are of vital importance in biomedical research and are often the best or only animal models for controlled experiments relevant to a range of human diseases and disorders. Macaque infection models provide unique opportunities for generating discoveries that may lead to new therapeutic options, improved vaccine strategies, and increased preparedness for future disease outbreaks. Among Old World monkeys, the pig-tailed macaques (*M. nemestrina*) were reported to be prone to HIV-1 infection with AIDS-like symptoms [26-29]. We and other groups identified TRIMCyp fusion protein expression in pig-tailed macaques. However, the fusion protein failed to restrict HIV-1 replication when expressed in some non-restrictive human or macaque cell lines [13-17].

Interestingly, the aforementioned pig-tailed macaques are divided into three macaque species according to current primate taxonomy [18]. Understanding the relationship between the *TRIM5 *locus and the susceptibility to HIV-1 in these species is urgently required. Here, we surveyed the *M. leonina *in Yunnan province, China. The results showed that the PBMCs of *M. leonina *are susceptible to HIV-1 infection, a finding which is consistent with previous results [13-17]. Moreover, the *M. leonina *fusion protein npmTRIMCyp can potently block HIV-2_ROD_, which may account for the different modulatory roles of host cell CypA, as the CypA is incorporated into HIV-1 but not HIV-2. Some other mechanisms may also exist, and one needs more work to dissect these possibilities. The same fusion pattern of *TRIM5-CypA *gene was observed in all the 11 surveyed *M. leonina *animals. Although it has been reported that both *M. nemestrina *and *M. mulatta *express TRIMCyp fusion proteins [15-17], our results suggest that the frequency of *TRIM5-CypA *fusion is higher in *M. leonina *than in *M. mulatta*. Further studies suggest that the fusion results from the insertion of the *CypA *pseudogene cDNA into the 3'-UTR of *TRIM5 *through the LINE-1-element-mediated retrotransposition. The transcription products of *TRIM5-CypA *were also detected in *M. leonina *PBMCs, and the transcript formation was attributed to the G-to-T substitution in the 3'-splicing site of *TRIM5 *intron 6 in macaques [17]. In the human genome, more than 60 processed CypA pseudogenes were reported across the genome by retrotransposition [30]. In New World primates, the *TRIM5-CypA *fusion gene was only identified in *Aotus *species, and the CypA exposed positive selection in the evolutionary history [31]. However, what drove the *CypA *pseudogene cDNA retrotransposition into the *TRIM5 *locus twice in the New World and Old World primates independently? These questions call for more genetic research to delineate the mechanistic details.

Host genetic variations have important impact on the susceptibility to HIV-1 infection. TRIM5α restricts HIV-1 through the B30.2/SPRY domain specifically recognizing and interacting with the CA protein. In owl monkey, the CypA copy in the TRIMCyp fusion protein can bind lentiviral CA and block their replication [11,12]. In pig-tailed macaques, *CypA *pseudogene cDNA substituted the B30.2/SPRY in the TRIM5α, resulting in the formation of the *TRIMCyp *fusion gene. Virgen *et al*. suggested that the TRIMCyp protein from *M. nemestrina *does not bind to HIV-1 CA because of the single amino acid (R69H) mutation in the TRIMCyp-CA interaction interface [15]. Interestingly, the npmTRIMCyp also contains the R69H mutation in the CypA domain, which may partly explain why the npmTRIMCyp cannot restrict HIV-1. The recognition domain of *M. leonina *TRIMCyp is relatively highly polymorphic. It would be interesting to analyze whether the specific polymorphism affects the sensitivity of the primates to HIV-1 infection. It might be possible to establish optimal HIV/AIDS NHP models in *M. leonina *through screening macaques that possess SNPs more susceptible to HIV-1 infection.

In conclusion, the *TRIM5-CypA *fusion in *M. leonina *maybe a pivotal factor associated with their susceptibility to HIV-1. The *M. leonina *appears to be a good candidate for an HIV/AIDS animal model. Our results should broaden the perspective in developing better HIV/AIDS NHP animal models.

## Materials and methods

### Animals

Northern pig-tailed macaques KMZ-1, KMZ-2, KMZ -4 and KMZ-5 were bred in the Kunming Zoo (KMZ), animals 524, 528, 551, 87015, 93201, 97203 and 99201 were raised in the Kunming Institute of Zoology (KIZ), Chinese Academy of Sciences (CAS) (Table 1). The Chinese rhesus macaque 95005 was raised in the KIZ, CAS. Whole blood from these animals was collected in EDTAK_2 _Blood Collection Tubes following the National Experimental Animal Handling Ordinance.

### RNA/DNA samples

PBMCs were isolated from EDTA K_2_-treated whole blood by EZ-Sep™ Monkey 9× (Dakewe Biotech) or Ficoll-Hypaque density centrifugation. Total RNA was isolated from 8 × 10^6 ^PBMCs using the RNAprep Cell Kit (Tiangen) following the manufacturer's instruction. Genomic DNA was isolated from 300 μl whole blood using the Puregene DNA Purification Kit (Qiagen) following the manufacturer's handbook.

### Genomic DNA Amplification and Sequencing

The PCR primers for amplifying the genomic DNA from intron 6 to the 3' flanking sequence (Table 2) were designed based on the sequences published in Genbank (accession numbers EU371641 and NT_009237). The fragments were amplified using LATaq-PCR or ExTaq-PCR kit (TaKaRa), purified with DNA Gel Extraction Kit (Watson Biotech), and cloned into the pMD 19-T Simple vector (TaKaRa), clones were picked up for sequencing analysis.

To screen for the G-to-T mutation associated with the 3'-splicing site within *TRIM5 *intron 6, the fragment encompassing the 3'-splicing site in intron 6 was PCR amplified with the sense primer T5in6F1 and the anti-sense primer T5ex8R3. The PCR products were digested with restriction endonuclease *Nsi *I (Fermentas), followed by electrophoresis in a 1.4% agarose gel [17]. In addition, the PCR products were purified and cloned for sequencing analysis.

### cDNA Amplification

Primers TRIMCypF and TRIMCypR (Table 2) for amplification of the complete coding sequence of Northern pig-tailed macaques *TRIM5 *have been described previously [13]. The total RNA was reverse transcribed into cDNA using the PrimeScript 1^st ^Strand cDNA Synthesis Kit (TaKaRa) following the manufacturer's instruction. The PCR condition was: 94°C for 2 minutes; 30 cycles of 94°C for 30 seconds, 55°C for 30 seconds, and 72°C for 1.5 minutes; held at 72°C for 7 minutes, and stored at 4°C.

### Sequence analysis

The sequences were assembled with the Contig program, and aligned by the Clustal X 1.83 or DNAStar 7.1.0 (Lasergene) software. Nucleotide sequence polymorphisms were analyzed by DnaSP 4.50 program. Neighbor-joining nucleotide tree of primate TRIMCyp was constructed via MEGA 3.1 program, bootstrap values were based on 1,000 replicates. Representative species of *Aotus trivirgirtas *was used as outgroup. The reference sequence accession numbers are: EU371639, EU371641, EU328216, AY646199, EU328216.

### Cell culture, transfection, and infection assay

Primates and human PBMCs were isolated by EZ-Sep monkey 9× or Ficoll-Hypaque PBMCs separation solution. PBMCs were cultured in RPMI 1640 complete medium supplemented with 5 μg/ml Phytohemagglutinin (PHA) (Sigma-Aldrich) and 50 IU/ml Interleukin-2 (IL-2) (Sigma-Aldrich) for 72 hours to activate the cells. Human T lymphocyte MT-4, monocyte U937, and a transformed Chinese rhesus macaque B lymphocyte line were cultured in complete RPMI 1640 medium. Activated PBMCs were seeded at the density of 5 × 10^5 ^cells/well in the 96-well plate, and the control cell lines were seeded at 4 × 10^5 ^cells/well. The HIV-GFP-VSVG viral stocks were thawed in a room temperature water bath, diluted with culture medium supplemented with 20 mM pH7.5 HEPES and 8 μg/ml polybrene (Sigma-Aldrich) on the ice. The cells were infected for 4 hours in the 37°C, 5% CO_2 _chamber, washed twice with phosphate-buffered saline (PBS), and then cultured in fresh RPMI 1640 complete medium. After 48 hours post-infection, the percentage of GFP positive cells was counted by FACS analysis using a FACSCalibur (Becton Dickinson).

HeLa-T4 cells were cultured in DMEM supplemented with 10% fetal calf serum (GIBCO), penicillin (Sigma), and streptomycin (Invitrogen). The C-terminally avian influenza Hemagglutinin (HA)-tagged TRIMCyp cDNA recombinant pLPCX expression plasmids of *M. leonina *and *A. trivirgatus *were constructed and transfected as previously described [13], npmTRIMCyp and omTRIMCyp expression in HeLa-T4 cells were detected by Western blot for the HA-tag, β-actin as a loading control. HeLa-T4 cells stably expressing TRIMCyp proteins were seeded in the 24-well plate at a density of 3 × 10^4 ^cells/well. On the following day, the cells were inoculated with viruses (MOI = 0.02) at 37°C for 2 hours. The cells were washed twice with PBS and cultured in 500 μl of fresh DMEM complete medium at 37°C, 5% CO_2_. Culture supernatants were harvested in duplicate on day 4 post-infection. The levels of HIV-2_ROD _and SIVmac239 capsid proteins in the medium were quantified by a p27-specific SIV p27 antigen ELISA kit (Zeptometrix).

### Nucleotide sequence accession numbers

The *M. leonina *TRIM5-CypA sequences are submitted for deposition in the GenBank database, the accession numbers are GQ180913–GQ180923.

## Competing interests

The authors declare that they have no competing interests.

## Authors' contributions

YTZ and YQK conceived of the study, and participated in its design. YQK, XT, and FLL carried out the experiments. YQK, YTZ, GG, XLJ and YPZ analyzed the results and drafted the manuscript. All authors read and approved the final manuscript.

## References

[B1] Simon F, Mauclère P, Roques P, Loussert-Ajaka I, Müller-Trutwin MC, Saragosti S, Georges-Courbot MC, Barré-Sinoussi F, Brun-Vézinet F (1998). Identification of a new human immunodeficiency virus type 1 distinct from group M and group O. Nat Med.

[B2] Gao F, Bailes E, Robertson DL, Chen Y, Rodenburg CM, Michael SF, Cummins LB, Arthur LO, Peeters M, Shaw GM, Sharp PM, Hahn BH (1999). Origin of HIV-1 in the chimpanzee Pan troglodytes troglodytes. Nature.

[B3] Keele BF, Van Heuverswyn F, Li Y, Bailes E, Takehisa J, Santiago ML, Bibollet-Ruche F, Chen Y, Wain LV, Liegeois F, Loul S, Ngole EM, Bienvenue Y, Delaporte E, Brookfield JF, Sharp PM, Shaw GM, Peeters M, Hahn BH (2006). Chimpanzee reservoirs of pandemic and nonpandemic HIV-1. Science.

[B4] Hofmann W, Schubert D, LaBonte J, Munson L, Gibson S, Scammell J, Ferrigno P, Sodroski J (1999). Species-specific, postentry barriers to primate immunodeficiency virus infection. J Virol.

[B5] Stremlau M, Owens CM, Perron MJ, Kiessling M, Autissier P, Sodroski J (2004). The cytoplasmic body component TRIM5α restricts HIV-1 infection in Old World monkeys. Nature.

[B6] Stremlau M, Perron M, Welikala S, Sodroski J (2005). Species-specific variation in the B30.2(SPRY) domain of TRIM5alpha determines the potency of human immunodeficiency virus restriction. J Virol.

[B7] Franke EK, Yuan HE, Luban J (1994). Specific incorporation of cyclophilin A into HIV-1 virions. Nature.

[B8] Thali M, Bukovsky A, Kondo E, Rosenwirth B, Walsh CT, Sodroski J, Göttlinger HG (1994). Functional association of cyclophilin A with HIV-1 virions. Nature.

[B9] Towers GJ, Hatziioannou T, Cowan S, Goff SP, Luban J, Bieniasz PD (2003). Cyclophilin A modulates the sensitivity of HIV-1 to host restriction factors. Nat Med.

[B10] Berthoux L, Sebastian S, Sokolskaja E, Luban J (2005). Cyclophilin A is required for TRIM5{alpha}-mediated resistance to HIV-1 in Old World monkey cells. Proc Natl Acad Sci USA.

[B11] Sayah DM, Sokolskaja E, Berthoux L, Luban J (2004). Cyclophilin A retrotransposition into TRIM5 explains owl monkey resistance to HIV-1. Nature.

[B12] Nisole S, Lynch C, Stoye JP, Yap MW (2004). A Trim-cyclophilin A fusion protein found in owl monkey kidney cells can restrict HIV-1. Proc Natl Acad Sci USA.

[B13] Liao CH, Kuang YQ, Liu HL, Zheng YT, Su B (2007). A novel fusion gene TRIM5-Cyclophilin A in the pig-tailed macaque determines its susceptibility to HIV-1 infection. AIDS.

[B14] Wilson SJ, Webb BL, Ylinen LM, Verschoor E, Heeney JL, Towers GJ (2008). Independent evolution of an antiviral TRIMCyp in rhesus macaques. Proc Natl Acad Sci USA.

[B15] Virgen CA, Kratovac Z, Bieniasz PD, Hatziioannou T (2008). Independent genesis of chimeric TRIM5-cyclophilin proteins in two primate species. Proc Natl Acad Sci USA.

[B16] Brennan G, Kozyrev Y, Hu SL (2008). TRIMCyp expression in Old World primates Macaca nemestrina and Macaca fascicularis. Proc Natl Acad Sci USA.

[B17] Newman RM, Hall L, Kirmaier A, Pozzi LA, Pery E, Farzan M, O'Neil SP, Johnson W (2008). Evolution of a TRIM5-CypA splice isoform in old world monkeys. PLoS Pathog.

[B18] Groves C (2001). Primate Taxonomy.

[B19] Rosenblum LL, Supriatna J, Melnick DJ (1997). Phylogeographic analysis of pigtail macaque populations (Macaca nemestrina) inferred from mitochondrial DNA. Am J Phys Anthropol.

[B20] Gippoliti S (2001). Notes on the taxonomy of *Macaca nemestrina leonina *blyth, 1863 (Primates: Cercopithecidae). Hystrix It J Mamm.

[B21] Li QQ, Zhang YP (2005). Phylogenetic relationships of the macaques (Ceropithecidae: Macaca), inferred from Mitochondrial DNA sequences. Biochem Genet.

[B22] Kazazian HH (2004). Mobile elements: Drivers of genome evolution. Science.

[B23] Vincent BJ, Myers JS, Ho HJ, Kilroy GE, Walker JA, Watkins WS, Jorde LB, Batzer MA (2003). Following the LINEs: An analysis of primate genomic variation at human-specific LINE-1 insertion sites. Mol Biol Evol.

[B24] Rozas J, Rozas R (1999). DnaSP version 3: an integrated program for molecular population genetics and molecular evolution analysis. Bioinformatics.

[B25] Rozas J, Gullaud M, Blandin G, Aguadé M (2001). DNA variation at the *rp49 *gene region of *Drosophila simulans*: Evolutionary inferences from an unusual haplotype structure. Genetics.

[B26] Agy MB, Frumkin LR, Corey L, Coombs RW, Wolinsky SM, Koehler J, Morton WR, Katze MG (1992). Infection of Macaca nemestrina by human immunodeficiency virus type-1. Science.

[B27] Kent SJ, Corey L, Agy MB, Morton WR, McElrath MJ, Greenberg PD (1995). Cytotoxic and proliferative T cell responses in HIV-1-infected *Macaca nemestrina*. J Clin Invest.

[B28] Bosh ML, Schmidt A, Chen J, Florey MJ, Agy M, Morton WR (2002). Enhanced replication of HIV-1 *in vivo *in pigtailed macaques (*Macaca nemestrina*). J Med Primatol.

[B29] Pekrun K, Shibata R, Igarashi T, Reed M, Sheppard L, Patten PA, Stemmer WP, Martin MA, Soong NW (2002). Evolution of a human immunodeficiency virus type 1 variant with enhanced replication in pig-tailed macaque cells by DNA shuffling. J Virol.

[B30] Zhang Z, Harrison PM, Liu Y, Gerstein M (2003). Millions of years of evolution preserved: a comprehensive catalog of the processed pseudogenes in the human genome. Genome Res.

[B31] Ribeiro IP, Menezes AN, Moreira MAM, Bonvicino CR, Seuánez HN, Soares MA (2005). Evolution of Cyclophilin A and *TRIMCyp *retrotransposition in New World Primates. J Virol.

